# Emerging threat of artemisinin partial resistance markers (*pfk13* mutations) in *Plasmodium falciparum* parasite populations in multiple geographical locations in high transmission regions of Uganda

**DOI:** 10.1186/s12936-024-05158-9

**Published:** 2024-11-05

**Authors:** Bosco B. Agaba, Jye Travis, David Smith, Simon P. Rugera, Maria G. Zalwango, Jimmy Opigo, Charles Katureebe, Ruth Mpirirwe, Dembo Bakary, Martin Antonio, Beshir Khalid, Joseph Ngonzi, Moses R. Kamya, Pontiano Kaleebu, Peter Piot, Qin Cheng

**Affiliations:** 1 The Medical Research Council Unit The Gambia at London School of Hygiene and Tropical Medicine: Peter Piot Fellowship for Global Health Innovation, Epidemic Preparedness & Response, Banjul, Fajara The Gambia; 2 Department of Medical Laboratory Sciences, Faculty of Medicine, Mbarara University of Science and Technology, Mbarara, Uganda; 3 National Malaria Control Division, Kampala, Uganda; 4 Infectious Diseases Research Collaboration, Kampala, Uganda; 5 QIMR Berghofer Medical Research Institute, Brisbane, Australia; 6 Australian Defence Force Malaria and Infectious Disease Institute, Brisbane, Australia; 7 World Health Organization, Country Office, Kampala, Uganda; 8 Makerere University, Kampala, Uganda; 9 London School of Hygiene and Tropical Medicine, London, UK; 10 Medical Research Council/London School of Hygiene and Tropical Unit, Uganda Virus Research Institute, Entebbe, Uganda

**Keywords:** Genomic surveillance, *Plasmodium falciparum*, *pfk13* mutations, Emergence, Artemisinin resistance

## Abstract

**Background:**

Artemisinin-based combination therapy (ACT) is currently recommended for treatment of uncomplicated malaria. However, the emergence and spread of partial artemisinin resistance threatens their effectiveness for malaria treatment in sub-Saharan Africa where the burden of malaria is highest. Early detection and reporting of validated molecular markers (*pfk13* mutations) in *Plasmodium falciparum* is useful for tracking the emergence and spread of partial artemisinin resistance to inform containment efforts.

**Methods:**

Genomic surveillance was conducted at 50 surveillance sites across four regions of Uganda in Karamoja, Lango, Acholi and West Nile from June 2021 to August 2023. Symptomatic malaria suspected patients were recruited and screened for presence of parasites. In addition, dried blood spots (DBS) were collected for parasite genomic analysis with PCR and sequencing. Out of 563 available dried blood spots (DBS), a random subset of 240 *P. falciparum* mono-infections, confirmed by a multiplex PCR were selected and used for detecting the *pfk13* mutations by Sanger sequencing using Big Dye Terminator method. Regional variations in the proportions of *pfk13* mutations were assessed using the chi square or Fisher’s exact tests while Kruskal–Wallis test was used to compare absolute parasite DNA levels between wild type and mutant parasites.

**Results:**

Overall, 238/240 samples (99.2%) contained sufficient DNA and were successfully sequenced. Three mutations were identified within the sequenced samples; *pfk13* C469Y in 32/238 (13.5%) samples, *pfk13* A675V in 14/238 (5.9%) and *pfk13* S522C in (1/238 (0.42%) samples across the four surveyed regions. The prevalence of *pfk13* C469Y mutation was significantly higher in Karamoja region (23.3%) compared to other regions, P = 0.007. The majority of parasite isolates circulating in West Nile are of wild type (98.3), P = 0.002. Relative parasite DNA quantity did not differ in samples carrying the wild type, C469Y and A675V alleles (Kruskal–Wallis test, P = 0.6373).

**Conclusion:**

Detection of validated molecular markers of artemisinin partial resistance in multiple geographical locations in this setting provides additional evidence of emerging threat of artemisinin partial resistance in Uganda. In view of these findings, periodic genomic surveillance is recommended to detect and monitor levels of *pfk13* mutations in other regions in parallel with TES to assess potential implication on delayed parasite clearance and associated treatment failure in this setting. Future studies should consider identification of potential drivers of artemisinin partial resistance in the different malaria transmission settings in Uganda.

## Background

Malaria remains a public health problem in Uganda. The country contributed 5.4% of the 249 million malaria cases reported globally making it the 3rd largest contributor to the global malaria burden in 2022 [1]. The entire country’s population remains at risk of infection. Transmission is highly heterogeneous between regions, sub-national levels and among specific sub-populations [2]. Uganda’s national malaria programme has achieved substantial success in implementation of the core interventions such as mass campaigns for long-lasting insecticidal nets (LLIN), indoor residual spraying (IRS), integrated community case management (iCCM), intermittent preventive treatment in pregnancy (IPTp) as well as case management. The programme has also invested substantially in the private sector in form of co-payments to subsidize anti-malarial drugs making them accessible to the population [2].

However, despite the investments and milestones achieved in intervention scale-up and coverage, the country’s progress against malaria has levelled-off and many districts and regions appear to be losing ground with potential for reversal [3]. Overall, the recent malaria programme review 2019–2020 has shown limited effect of interventions on reducing malaria burden in all districts. Recently, malaria control in Uganda is further threatened by the reports of emerging biological and genetic changes in malaria parasites and vectors. While artemisinin-based combination therapy (ACT) remains efficacious against malaria, presence of partial artemisinin resistance has been reported for the first time on the African continent with reports of delayed parasite clearance. [4, 5]. Continuous monitoring of ACT efficacy is needed to inform treatment policies in malaria-endemic countries, and to ensure early detection of, and response to, drug resistance [6–8].

Uganda’s Malaria Programme adopted the World Health Organization (WHO) recommendation on the use of ACT as the first- and second-line treatment for uncomplicated *Plasmodium falciparum* malaria [9, 10]. The country’s current treatment policy for uncomplicated malaria recommends three artemisinin-based combinations that combine an artemisinin derivative (artesunate, artemether and dihydroartemisinin) with a partner drug (amodiaquine, lumefantrine and piperaquine), respectively. The role of the artemisinin compound is to reduce the number of parasites during the first 3 days of treatment (reduction of parasite biomass), while the role of the partner drug is to eliminate the remaining parasites (cure). Artemisinin partial resistance first emerged in Cambodia as delayed parasite clearance after treatment with artemisinin derivatives and increased survival of ring stage parasites following exposure to artemisinin derivatives [9]. In vitro and in vivo studies have shown that mutations in the *Pf*Kelch13 propeller domain (*pfk13* gene) are associated with this delayed parasite clearance [8, 9]. The molecular markers based on mutations of the *pfk13* gene are classified as either validated, associated or candidate markers. In order to classify a marker as a candidate or associated *pfk13* markers of artemisinin partial resistance, there must be a statistically significant association (p < 0.05) between that *pfk13* mutation and parasite clearance half-life > 5 h, or day 3 parasitaemia on a sample of at least 20 clinical cases, or survival of > 1% using the RSA^0–3 h^ in at least five individual isolates with a given mutation, or a statistically significant difference (p < 0.05) in the RSA^0–3 h^ assay between culture-adapted recombinant isogenic parasite lines, produced using transfection and gene editing techniques, which express a variant allele of *pfk13* as compared with the wild-type allele. The *pfk13* mutations are classified as a validated marker of artemisinin partial resistance if it satisfies both of the above requirements [11].

Particularly more concerning are recent reports of emergence of artemisinin partial resistance in Africa particularly in Rwanda, Uganda, Tanzania and the Horn Africa [5, 8, 12–15].

Available evidence shows that the selection of *pfk*13 mutations in Uganda was at a comparable rate to that observed in South-East Asia (SEA) in samples collected between 2016 and 22, suggesting that *pfk*13 mutations may continue to increase quickly in Uganda [16]. Several studies previously conducted in Uganda have reported the presence of *pfk13* 469Y, C469F, P574L and A675V as common mutations in Northern and other regions of Uganda [14, 17] while others reported non-synonymous and synonymous p*fk13* single nucleotide polymorphisms (SNPs) [18, 19]. Other studies either reported lower prevalence or detected no *pfk*13 mutations in Ugandan parasite isolates [20–26]. Among the *pfk13* mutations reported in previous studies in Uganda, the C469Y, A675V and P574L are all classified by the WHO as validated markers of partial artemisinin resistance. Recent therapeutic study in Uganda (unpublished 2023) has shown decreased artemether/lumefantrine efficacy of 84.6% (95% CI 76.5–90.3) and 82.9% (95% CI 74.5–88.9) at Busia and Arua sites, respectively.

Elsewhere on the African continent similar markers of resistance have been reported in Rwanda, Sudan, Tanzania, Ethiopia and Eritrea suggesting widespread potential for artemisinin resistance in parasite population on the continent [15, 27–30]. Previous reports suggest independent and spontaneous emergence of parasites with *pfk13* mutations in multiple location based on evidence of unrelatedness between mutant strains collected in different geographical locations [13, 31]. Delayed parasite clearance has been detected in returning travellers in the UK and other non-malaria endemic territories posing threat to global health security [32–34]. In an effort to manage and contain the threat, the WHO has recently developed a strategy for artemisinin resistance management for Africa [8]. Due to the changing dynamics of malaria transmission and parasite related biological changes, the molecular epidemiology and the extent of spread of artemisinin resistant parasites as well as its effect on malaria control in northern regions of Uganda is unclear. Widespread artemisinin resistance, as observed in Southeast Asia, has the potential to disrupt and reverse significant gains that have been achieved against the malaria burden in Africa [8, 35, 36]. Timely molecular surveillance and detection of *pfk13* mutations is a useful tool for tracking the emergence and containment of the spread of artemisinin resistance [10, 11]. The purpose of this study was to assess the presence and distribution of *pfk13* polymorphisms in *P. falciparum* parasites at fifty surveillance sites in Northern Uganda representing a wide range of transmission setting.

## Methods

### Study design and setting

A total of 563 dried blood spots (DBS) were available from a primary study for *pfhrp2* and *pfhrp3* deletion surveillance [37]. In the primary study, all the 563 available DBS samples had been confirmed with multiplex qPCR for presence of *P. falciparum* parasite DNA out of which 73.9% (416/563) gave valid PCR results. The current study utilized a random sample of 240 dried blood spots (selected from the 416) that were sequenced to assess the presence of *pfk13* mutations across the surveillance regions. Selection of 240 DBS for sequencing ensured even distribution across the four survey regions (60 per region).

**Fig. 1:**
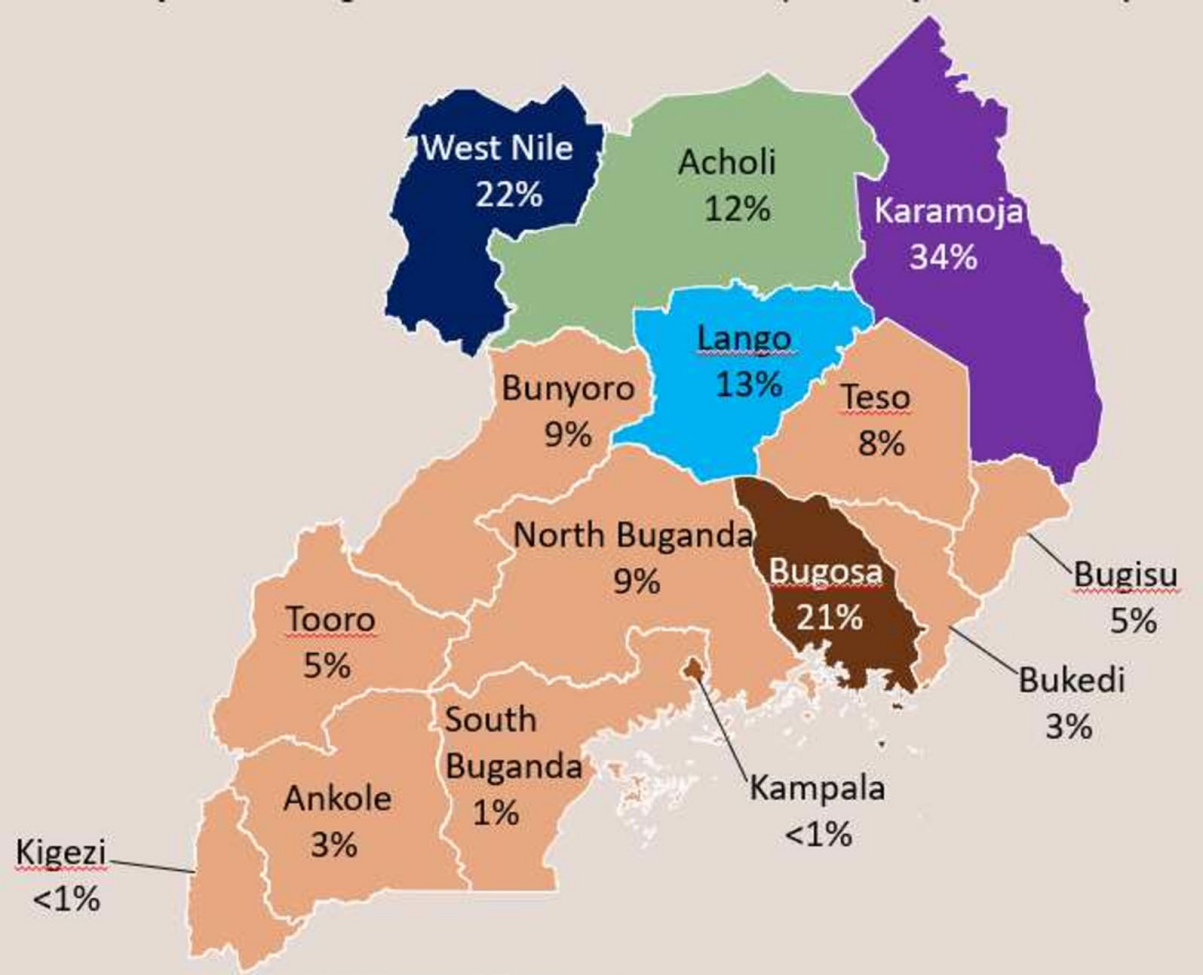
Malaria transmission and parasite prevalence in the survey regions. Figure shows the population-based parasite prevalence per survey region including the four regions of Karamoja, Lango, Acholi and West Nile from where filter papers samples were collected (The figure is extracted from the most recent Uganda national malaria indicator survey report [38])

Details of the study population, settings, DBS collection and sampling were published under the primary study where the samples originated [37]. Briefly, capillary blood was collected from malaria symptomatic patients seeking care across 50 surveillance sites in the regions of Acholi, Karamoja, W. Nile and Lango in northern Uganda. Capillary blood collected from eligible patients screened and enrolled was spotted onto filter paper (Whatman No. 903). The four regions are well designed demographic health survey (DHS) clusters or enumeration areas that are periodically used for the national malaria indicator surveys in the country [38] and serve a total population of 10 million people that are at risk of malaria infection based on the recent national housing and population census [39]. Malaria transmission across all the four regions is intense and stable at holoendemic levels, with a test positivity rate (TPR) ranging from 50 to 70% at health facility and parasite prevalence of 13–34% as determined by blood smear microscopy (Fig. 1) [38].

### Parasite DNA extraction and quality control

Genomic DNA extracted previously [37] for the detection of *pfhrp2/3* deletions was stored at − 20 °C to preserve its integrity and was available for use in this study to amplify and sequence for *pfk13* mutations. The presence of parasite DNA in the samples was confirmed using a multiplex qPCR assay adapted from Grignard et al*.* [40], which detects human tubulin, parasite lactate dehydrogenase (pLDH), and *P. falciparum* histidine rich proteins 2 (*pfhrp2*) and 3 (*pfhrp3*) genes. Samples positive for both human tubulin and pLDH were deemed eligible for selection for *pfk13* analysis, regardless of their *pfhrp2* and *pfhrp3* status. Quantity of parasite DNA relative to human blood volume in each sample was calculated based on ΔCq values of pLDH-human tubulin as an indicator for parasite density.

### PCR amplification of *pfk13* propeller region

Amplification of the *pfk13* propeller region (849 bp (1329-to-2178 nucleotides) was achieved through nested PCR as described previously by Ariey et al*.* [41] using primers K13-1 5′-cggagtgaccaaatctggga-3′ and K13-4 5′-gggaatctggtggtaacagc-3′ in the primary PCR as well as K13-2 5′-gccaagctgccattcatttg-3′ and K13-3 5′-gccttgttgaaagaagcaga-3′ in the nested PCR. Both the primary and nested PCR reactions consisted of 1 μl of DNA template, 1 μM of each primer, 0.2 mM dNTPs (Invitrogen, USA) and 2 U Taq DNA polymerase (Invitrogen, USA) in a volume of 25 μl. The cycling programme was as follows: 5 min at 94 °C, followed by 40 cycles of 30 s at 94 °C, 90 s at 60 °C, 90 s at 72 °C and final extension of 10 min at 72 °C on a Mastercycler X50s (Eppendorf, Germany).

### Sequencing of *pfk13* propeller region

PCR products were confirmed using gel electrophoresis on a 2% agarose gel stained with GelRed® Nucleic Acid Gel Stain (Biotium, USA). Positive samples were purified with the DNA Clean & Concentrator-5 PCR purification kit (Zymo, USA) and sequenced from both directions. A mixture containing 3 μl purified PCR product, 2 μl unidirectional primer (K13-2 or K13-3) and 5 μl nuclease-free water was submitted to QIMR Berghofer Medical Research Institute (Brisbane, Queensland, Australia) for sequencing with Big Dye Terminator v3.1 (Applied Biosystems, USA). Sequences were aligned using ClustalW in MEGA 11 with the corresponding region from parasite clone *Pf*3D7 used as a reference. Sequencing trace of samples that had both wild type and mutant sequence peaks at the same location were classified as mixed wild type and mutant alleles. Proportions of mutant in a sample was determined by peak height of mutant allele relative to wild type allele as 10–29%, 30–59%, 60–89% and 90–100%. Mutant allele was determined as a dominant or non-dominant allele when its peak height relative to wild type was > 60% or < 60%, respectively.

### Data analysis

Electronic excel database of the previous study that linked study identification numbers of samples to their respective demographics was accessed for data extraction. Data quality checks were done to assess its quality and consistency. Data analysis was done with STATA Version 14 (College Station, TX, USA: StataCorp LP). Descriptive analysis was done to describe the baseline characteristics of samples and determine proportion, distribution and spread of *pfk13* mutations in the four surveillance regions. Statistical analysis was done using Chi-square or Fisher’s exact test to assess regional variation in proportions of *pfk13* mutations while Kruskal–Wallis test was used to compare absolute parasite DNA levels between wild type and mutant parasites with a p value < 0.05 considered statistically significant.

### Ethical approval

The study was approved by the Makerere University School of Public Health Research and Ethics committee (#REC REF SPH-2021-135) and the Uganda National Council of science and technology (Ref No. HS1911ES). In the study surveys from where samples were collected, participants provided written informed consent for future use of biological samples for molecular analysis.

## Results

### Baseline characteristics of dried blood spots from where the sequenced samples originated

Overall, 390 (69.4%) and 173 (30.6%) were from symptomatic individuals of ≥ 5 and < 5 years old respectively. Samples were evenly distributed across all the four regions of Karamoja 24.0% (135), West Nile 23.7% (133), Acholi 24.8% (140) and Lango 27.4% (155) Table 1. Across the four surveillance regions, a total population of 10,000,000 people is at risk of malaria infection.

**Table 1:** Characteristics of samples (N = 563) from where the K13 sequenced samples originated

Variables	Frequency (n)	Proportion (%)
Age (years)		
< 5	173	30.6
≥ 5	390	69.4
Sex		
Male	220	39.0
Female	343	60.7
Samples collected per survey region		
Acholi	140	24.9
Lango	155	27.4
W. Nile	133	23.7
Karamoja	135	24.0

### Parasite DNA amplification and *pfk13* mutations detected in the samples

Out of a random sample of 240 PCR positive DBS, 238/240 (99.2%) were successfully sequenced and returned good sequences. Three (mutations) polymorphisms in the *pfk13* propeller region were identified in these 238 samples: C469Y, S522C and A675V. Mutation S522C was detected in only one sample from Lango (1/238, 0.42%). The most prevalent mutation, C469Y, was detected in 32/238 samples (13.6%), and majority (31/32) were detected in Acholi, Karamoja and Lango regions. Furthermore, 21/32 (65.6%) of the samples with C469Y mutation had mixed sequences of both the wild type and mutant alleles. 11/15 (73.3%) of samples where the mutant is dominant (> 60%) originated in Karamoja, whereas non-dominant (< 60%) mutations were more common in Lango (8/9, 88.9%) and in Acholi (5/8, 62.5%). *pfk13* A675V was detected in 14/238 (5.9%) valid sequences which were spread evenly across Acholi (4/14, 28.6%), Karamoja (5/14, 35.7%) and Lango (5/14, 35.7%). The wild type allele only was detected in 191/238 samples (80.3%), majority of which were in West Nile. An additional 21 samples (21/238, 8.8%) contained mixed *pfk13* C469Y mutations i.e. both the wild type and mutant alleles were detected (Table 2).

**Table 2:** Prevalence and distribution of the PfK13 mutations (S522C, A675V, C469Y) and C469Y mutant allele or mixed with wild-type allele across the survey regions

Region	Valid sequences	Wild Type Only		S522C		A675V		C469Y^a^		C469Y							
										10–29%^b^		30–59%^b^		60–89%^b^		90–100%^c^	
		n	%	n	%	n	%	N	%	n	%	N	%	n	%	n	%
Acholi	59	47	79.7	0	0	4	6.8	8	13.6	5	8.5	0	0.0	1	1.7	2	3.4
West Nile	59	58	98.3	0	0	0	0.0	1	1.7	0	0.0	1	1.7	0	0.0	0	0.0
Karamoja	60	41	68.3	0	0	5	8.3	14	23.3	2	3.3	1	1.7	3	5.0	8	13.3
Lango	60	45	75.0	1	1.7	5	8.3	9	15.0	3	5.0	5	8.3	0	0.0	1	1.7
Total	238	191	80.3	1	0.4	14	5.9	32	13.4	10	4.2	7	2.9	4	1.7	11	4.6

^a^Includes mixed parasite populations with C469Y mutant allele and wild-type alleles

^b^% mutant in sample

^c^Prevalence of single clone mutation

### Regional variation of *pfk13* mutations

The *pfk13* mutation C469Y was higher in proportion in Karamoja region (23.3%) compared to other surveillance regions (P = 0.007). The largest proportion of parasite populations circulating in West Nile was of wild type (58/59, 98.3%), significantly higher than other regions (P = 0.002). In contrast, the proportions of the S522C and A675V were not significantly different across the study regions (Table 3).

**Table 3:** Regional variation of Pfk13 mutations

Mutation	Status	Acholi (n = 59) (%)	Karamoja (n = 60) (%)	Lango (n = 60) (%)	West Nile (n = 59) (%)	P value
Wild Type	Present	47 (79.6)	41 (68.3)	45 (75.0)	58 (98.3)	0.002^$^
	Absent	12 (20.3)	19 (31.7)	15 (25.0)	1 (0.0)	
C469Y	Present	8 (13.6)	14 (23.3)	9 (15.0)	1 (1.7)	0.007^$^
	Absent	51 (86.4)	46 (76.7)	51 (85.0)	58 (98.0)	
S522C	Present	0 (0.0)	0 (0.0)	1 (1.7)	0 (0.0)	1.000*
	Absent	59 (100.0)	60 (100.0)	59 (98.3)	59 (100.0)	
A675V	Present	4 (6.8)	4 (6.7)	5 (8.3)	0 (0.0)	0.095*
	Absent	55 (93.2)	56 (93.3)	55 (91.7)	59 (100.0)	

^$^Chi square P value, *Fishers exact test P value

### Relative quantity of parasite DNA

**Fig. 2:**
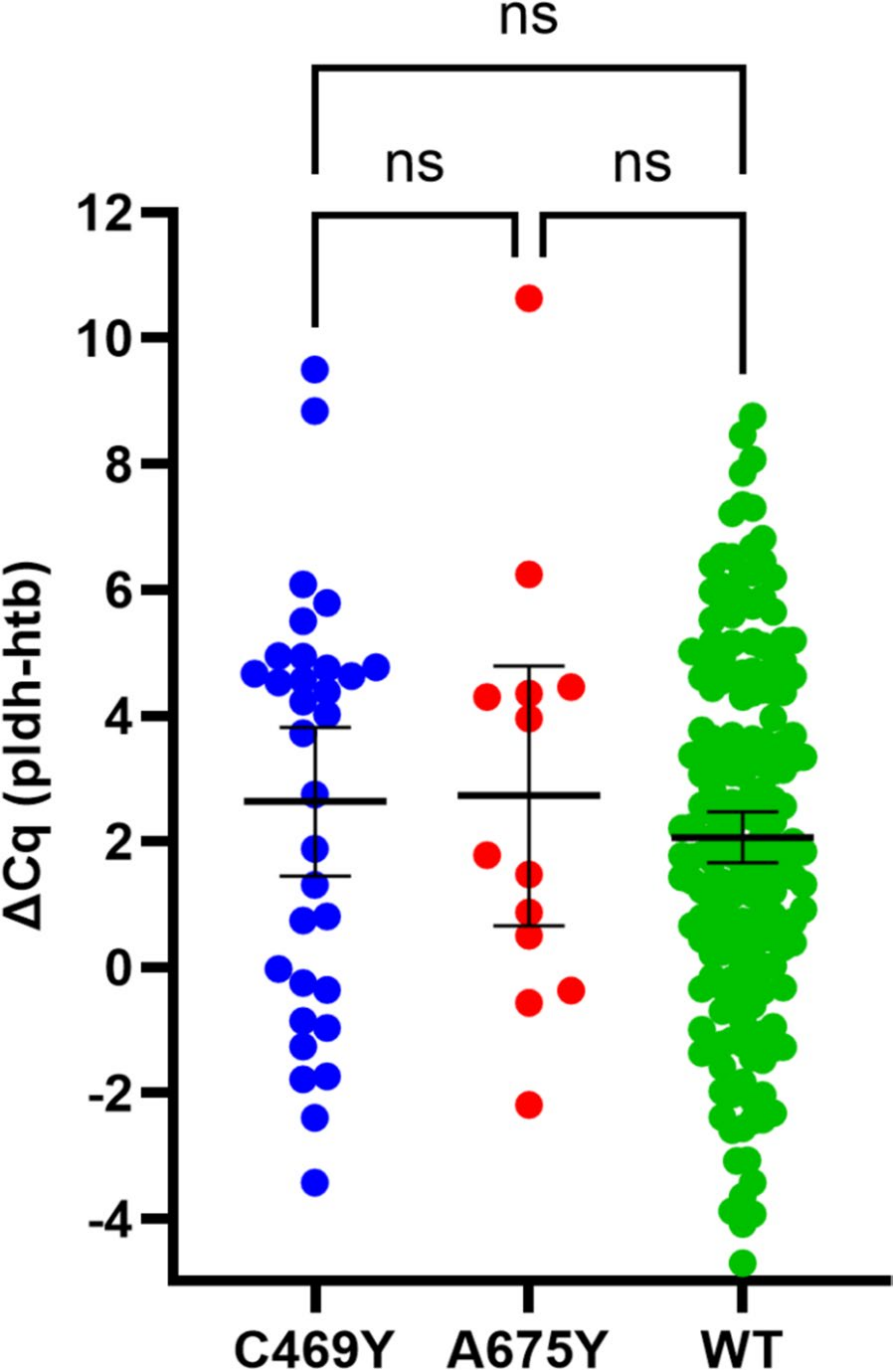
Comparison of relative parasite DNA quantity between wild type (WT) samples and those with C469Y or A675V mutations. *Ns* not significant, *wt* wild type, *pldh* parasite lactate dehydrogenase, *cq* quantification cycle (cycle number at which the curve first rises above the threshold level), *htb* human tubulin

Relative parasite DNA quantity (as a surrogate for parasite density in peripheral blood) in samples carrying the wild type, C469Y and A675V alleles was compared between wild type and mutants based on ΔCq (pLDH-Human tubulin). There was no significant difference (Kruskal–Wallis test, p = 0.6373) in DNA concentrations across the three *pfk13* alleles (WT, C469Y, A675V) suggesting comparable peripheral parasite densities between parasites carrying a wild type and a mutant *pfk13* (Fig. 2).

## Discussion

Genomic surveillance data for malaria parasites and vectors can provide key information for implementation and control efforts in malaria endemic regions including timely detection and response to biological threats such as the development of resistance to drugs and insecticides. Molecular markers of partial artemisinin resistance (*pfk13* mutations) have been used as early pointers of partial artemisinin resistance to inform early response and containment efforts [8]. Historically, Uganda introduced and adopted the use of ACT as treatment of choice for uncomplicated malaria in early 2000’s. Reports of development of partial artemisinin resistance are coming on board close to two decades after the initial introduction of ACT in Uganda. The detection of validated markers of artemisinin resistance *pfk13* C469Y and *pfk13* A675V in Northern Uganda adds to the existing body of evidence reporting potential development of partial artemisinin resistance in Africa [8, 13]. Similarly, the C469Y and 675V had been previously reported in Central region of Uganda [14]. On the contrary, different types of *pfk13* mutations 469F and 561H were detected at high prevalence in southwestern Uganda near the Uganda-Rwanda border [14, 26]. The 561H had been detected at high prevalence in Rwanda where partial artemisinin resistance has been confirmed [15, 42]. The P441L was also detected at several sites in far western Uganda [14].

Of concern is the presence of validated molecular markers of partial artemisinin resistance in this traditionally high malaria transmission setting in northern Uganda. Validated makers are known to improve parasite survival in vitro during the ring-stage survival assay (RSA*)*, as well as delayed parasite clearance in vitro or in clinical cases assessed by day 3 parasitaemia [43]. The *pfk13* mutations, such as C580Y and R539T, have been associated with partial artemisinin resistance in the greater Mekong region where the first reports of delayed parasite clearance and treatment failure following ACT occurred [44, 45]. Uganda’s malaria treatment policy recommends the use of artemether in combination with lumefantrine as partner drug for first line treatment of uncomplicated malaria, Artesunate combined with amodiaquine as alternative first line treatment for uncomplicated malaria and dihydroartemisinin combined with piperaquine as partner drug as second line treatment for uncomplicated malaria. The drugs were selected based on evidence on safety and efficacy generated from in-country therapeutic efficacy studies [10]. Although artemisinin resistance data in Uganda is currently limited by coverage, delayed parasite clearance has been reported in therapeutic efficacy studies (TES) at Arua site in West Nile and Busia site in Eastern Uganda [46], which points to potential development of resistance in the country.

The observed heterogeneity in the frequency of *pfk13* mutations between regions may suggest differences in the intrinsic factors linked to the parasite, host and drugs used and the environmental factors all of which are documented drivers of artemisinin resistance. Treatment practices (both patient and provider) related drivers include those affecting frequency dose and duration in which parasite population is exposed to the given drug [8]. However, additional studies need to be conducted to establish the actual drivers of partial artemisinin resistance in the Ugandan context. While the malaria treatment policy on first- and second-line drugs for the treatment of uncomplicated and severe malaria applies uniformly in Uganda, previous surveys by ACT Watch have reported the presence and use of unauthorized artemisinin-based combinations circulating in the private sector including monotherapies [47, 48]. Additional ACT surveys conducted under the Affordable Medicines Facility for malaria (AMFm) co-payments have reported similar findings of presence of non-quality assured ACT on the market in Uganda and Tanzania [48, 49]. Unauthorized and non-quality assured ACT are associated with poor quality based on the active pharmaceutical ingredient (API) of artemisinin measurements that are outside the acceptable WHO API range and the associated risks of sub-optimal doses [49]. Based on these survey findings and those from previous studies, it can be seen that the Northern region particularly Acholi and Lango accounts for the higher burden of validated ACT resistance markers [5, 14, 21]. Additional plausible explanation for this trend could be related to the political instability that lasted close to two decades in those regions characterized by population displacements [50]. Political instability is associated with breakdown of health systems affecting health service delivery such as implementation of case management trainings, treatment policies as well as supervision of drug use and regulation both in public and private sector [51]. Uncontrolled drug use and unregulated antimalaria drug supply chains can potentially continuously expose the parasite to sub-optimal doses and hence mounting intense selection pressure leading to resistance. Similar conditions existed in the Mekong region particularly at the Thai-Cambodia border that has been described as the epicenter and origin of all anti-malarial drug resistance [52, 53]. The area is characterized by uncontrolled cross border movements of forest and rubber plantation workers reported to have contributed to chloroquine and later artemisinin resistance in the region [54].

Interestingly, the prevalence of *pfk13* C469Y was higher in Karamoja region (23.3%) where malaria transmission is persistently highest over the last 5 years based on the last malaria indicator surveys [38]. The association between presence of molecular markers and the persistent malaria transmission in the Karamoja region needs to be investigated further including consideration for positioning a TES site. Historically, the Karamoja region in inhabited by mobile nomadic and pastoralist populations with uncontrolled activities across the porous border between Karamoja region and North western Kenya which could provide potential favorable conditions for drug resistance development [55]. Similarly, the presence of mutations C469Y and A675V in relative higher frequency in Lango and Acholi may suggest close monitoring to detect any delayed parasite clearance including pharmacokinetic studies to monitor efficacy of the partner drugs. A single sample carrying S522C was identified in Lango indicating its low frequency in the study areas. This mutation had been reported to be associated with delayed parasite clearance, but not considered by the WHO as a validated or candidate mutation due to insufficient evidence [56] and was detected in low frequency in Kenya [57].

Finally, the impact of *pfk13* mutations on parasite fitness remains unclear. While C580Y mutation was shown to confer modest fitness costs when accompanied by multicopy *pm2/3*, it remains fitness-neutral in the presence of single *pm2/3* copy [58]. An assessment was done as part of the current study to check if the parasite isolates carrying the *pfk13* mutations had different DNA levels (a proxy of parasite densities) in peripheral blood of patients compared to parasites with wild type *pfk13* alleles. A significant difference in parasite densities may provide an indication if *pfk13* mutant parasites have likely gained or lost fitness compared to the wild type parasites. Similarities in relative DNA concentrations across the wild type, C469Y and A675V *pfk13* alleles between the parasites (Kruskal–Wallis test, p = 0.6373) indicated comparable peripheral parasite densities between parasites carrying a wild type and a mutant *pfk13* suggesting comparable parasite fitness during blood stage. Future surveys could consider parasite density and fitness studies between wild type and mutant parasites (with K13 mutations) in longitudinal prospective designs.

### Implication for the national malaria programme

The presence of parasites with validated markers of partial artemisinin resistance particularly in this traditionally high malaria transmission setting calls for increased vigilance that may include development of a country specific artemisinin resistance management strategy. Expansion of parasite genomic surveillance to enhance detection of molecular markers of artemisinin resistance in other regions of Uganda as well as monitoring the levels in regions where they have been confirmed is recommended [8]. In areas with confirmed presence of *P. falciparum* parasites with validated markers of partial artemisinin resistance, consideration for location and positioning of TES sites may be necessary to assess the extent to which presence of markers at those levels translates into delayed parasite clearance or treatment failure in this setting. In view of these findings and similar results reported previously [5, 14, 21, 26, 46, 59, 60], the programmatic implication may include comprehensive evidence review to identify the possible drivers of partial artemisinin resistance in Uganda to inform containment efforts. Based on the current guideline [8], the country specific strategy would aim to intensify genomic surveillance and TES to enhance early detection, delay the emergence of resistance through appropriate treatment practices and drug regulations to avoid exposure of the parasites to sub-optimal doses of ACT and finally, to limit the spread of resistant parasite strains to other areas. For now, only one TES has reported declining efficacy of artemether/lumefantrine at Busia and Arua study sites with PCR-corrected efficacy of AL below the 90% WHO threshold at both sites [46]. The declining efficacy seen from TES coupled with presence of markers calls for increased vigilance to detect and contain emergence of partial artemisinin resistance that could be a potential disaster for malaria control efforts.

## Conclusion

Detection of validated molecular markers of artemisinin partial resistance in multiple geographical locations in this high malaria transmission setting provides additional evidence of emerging threat of partial artemisinin resistance in Uganda. In view of these findings, periodic genomic surveillance is recommended to detect and monitor levels of *pfk13* mutations in other regions in parallel with TES to assess potential implication on delayed parasite clearance and associated treatment failure in this setting. Future studies should consider identification of potential drivers of artemisinin partial resistance in the different malaria transmission settings in Uganda.

## Data Availability

No datasets were generated or analysed during the current study.

## Abbreviations

ACT: Artemisinin-based combination therapy
Cq: Quantitative cycle
DBS: Dried blood spots
DHS: Demographic health program
HRP2: Histidine rich protein 2
htb: Human tubulin
PCR: Polymerase chain reaction
*pfhrp2*: *Plasmodium falciparum* histidine rich protein 2 gene
*pfhrp3*: *Plasmodium falciparum* histidine rich protein 3 gene
pldh: Parasite lactate dehydrogenase
qPCR: Quantitative polymerase chain reaction
RDTs: Rapid diagnostic tests
RSA: Ring-stage survival assay
TES: Therapeutic efficacy studies
WHO: World Health Organization
WT: Wild type
